# Primary dural lymphoma: Case report

**DOI:** 10.1016/j.amsu.2022.103984

**Published:** 2022-06-11

**Authors:** Jihane Saidy, Abderrazzak Bertal, Saad Hmada, Nidal Aamara, Yassine Tahrir, Sara Mokhliss, Mohamed Karkouri, Abdessamad Naja, Abdelhakim Lakhdar

**Affiliations:** Neurosurgery Department, University Hospital Center IBN ROCHD, Casablanca, Morocco

**Keywords:** Primary central nervous system lymphomas, Primary dural lymphomas, Case report

## Abstract

Primary Dural lymphoma (PDL) is a rare pathology that occurs in immunocompetent patients. In such cases, these lesions may mimic more common intracranial tumors. We present the case of a patient who presented an intra cranial hypertension syndrome; the brain MRI showed a tissular mass that we took for a meningioma; upon surgical intervention, an occult mass was discovered. Major excision and immunohistochemistry demonstrated PDL. Our case report highlights the rarity of these pathology and the importance of combined surgery and medical treatment, as the latter can be treated with chemoradiation with good clinical outcomes.

## Highlights

• Primary dural lymphoma (PDL) is a rare pathology that occurs in immunocompetent patients.
• We present the case of a patient who presented an intra cranial hypertension syndrome; the brain MRI showed a tissular mass that we took for a meningioma; upon surgical intervention, an occult mass was discovered.
• Our case report highlights the rarity of these pathology and the importance of combined surgery and medical treatment.

## 1 Introduction

**Fig. 1:**
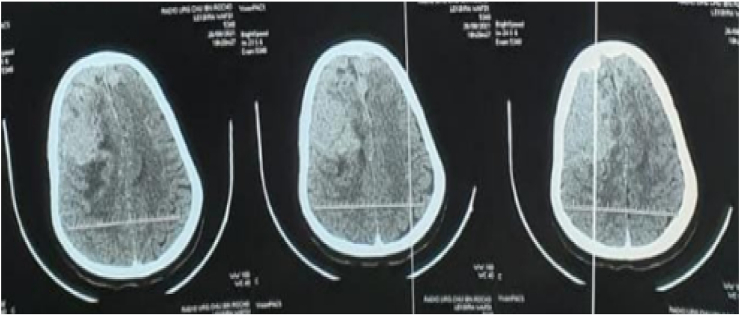
CT-scan was performed objectifying a right frontal extra axial process with extension at the left frontal level intensively taken the contrast medium.

**Fig. 2:**
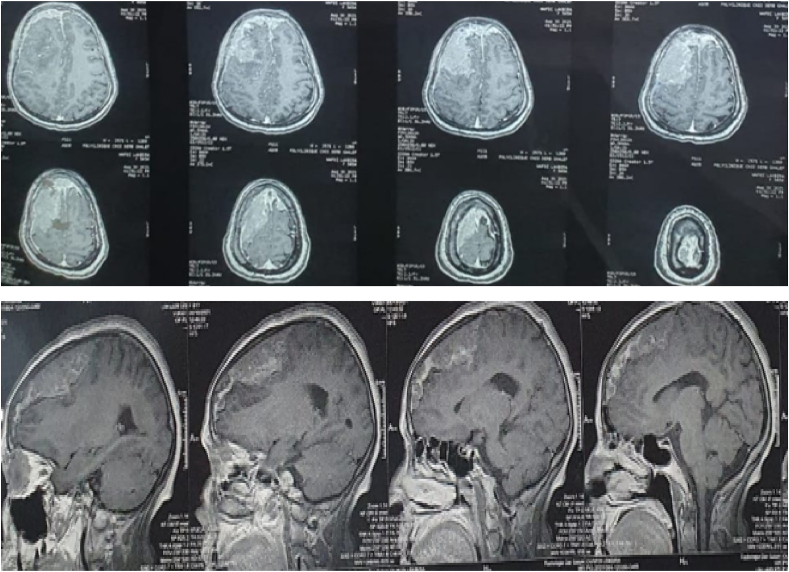
A brain MRI showed the same image with dural attachment on the right frontal lobe.

**Fig. 3:**
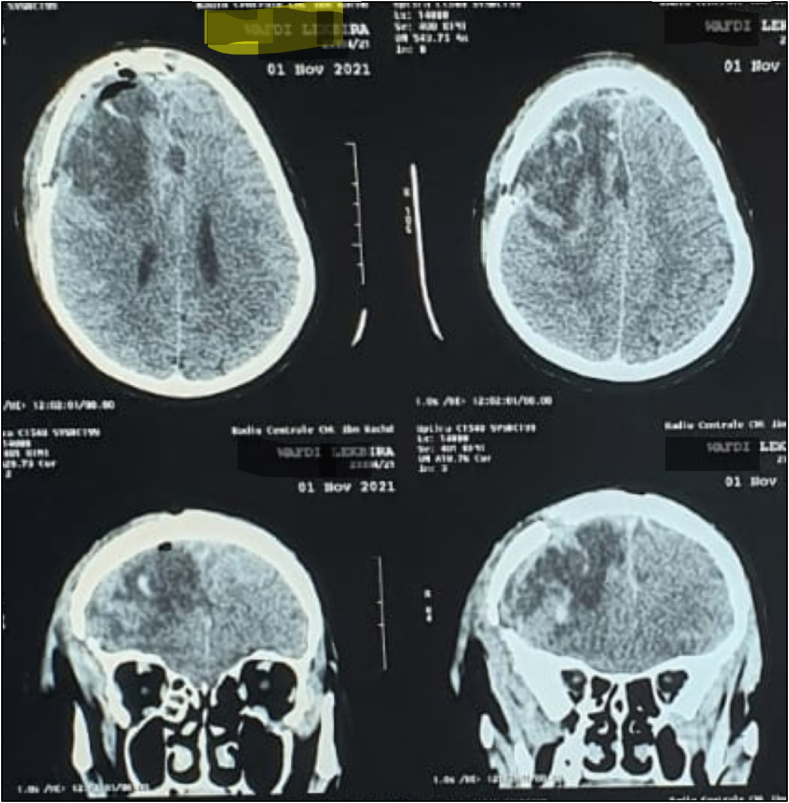
Showing the complete removal of the tumor in control CT scan.

**Fig. 4:**
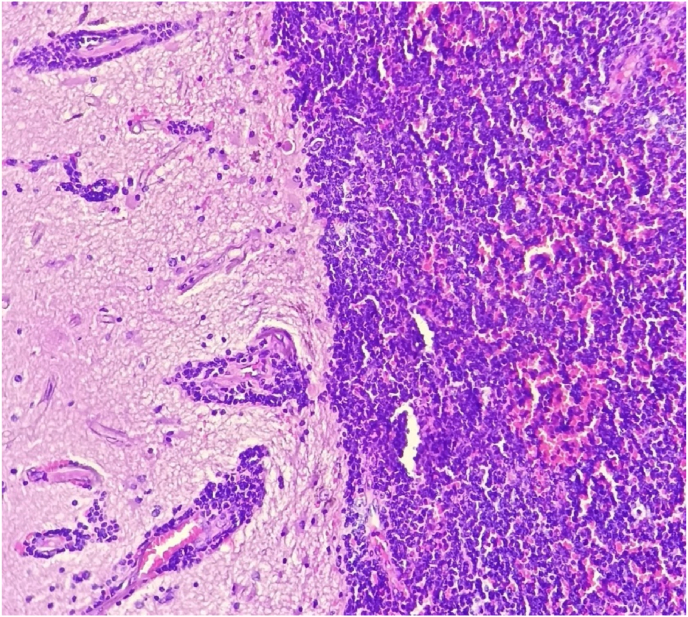
Massive cerebral infiltration by a lymphoproliferation with small cells arranged in a sheet describing by place perivascular sleeves.

**Fig. 5:**
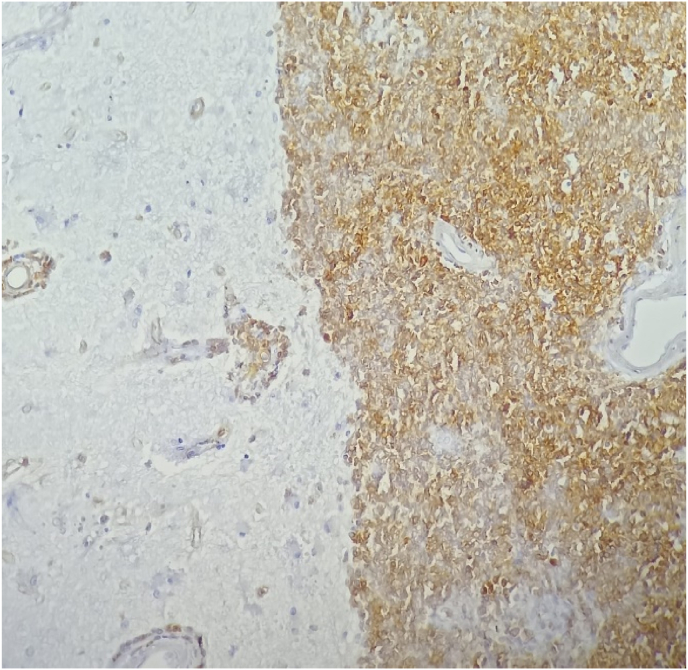
Immunohistochemical analysis showing diffuse expression of CD20 by tumor proliferation.

Primary central nervous system lymphoma (PCNSL) is a rare variant of non-Hodgkin's lymphoma (NHL), which is restricted to the brain, leptomeninges, cranial nerves, spinal cord, or intraocular compartment without involvement of other organ systems [3]. Primary dural lymphoma (PDL) is a subtype arising from this larger group. This pathology is accounted for less than 1% of all central nervous system (CNS) lymphomas and <0.1% of all non-Hodgkin's lymphomas generally [1,6]. Based on the site of arising the lymphomas can be divided nodal or extra nodal. In this observation we report a case of primary dural lymphoma in immune competent patient revealed by a intra cranial hypertension (see Fig. 1, Fig. 2, Fig. 3, Fig. 4, Fig. 5).

## 2 Case report

Our patient is 61 years old with no pathological history. Her symptoms began with an inaugural seizures 2 months before her admission associated to headaches, without any other symptoms.

The clinical examination did not find anything in particular, in particular no sensory-motor deficit or damage to the cranial nerves. Optical examination was also normal.

The surgical intervention was programmed, and a subtotal excision was realized with our professor. The tumor attachment was on falx, and it was fibrous non-vacuumable.

The post operative follow-up simple without hemorrhagic complications and without the occurrence of a deficit. The patient was transferred to hematology department.

This case has been reported in line with the 2020 SCARE guidelines [7].

## 3 Discussion

Primary dural lymphoma (PDL) is a subtype arising from a larger group called primary central nervous system lymphomas (PCNSL). PCNSL are extra nodal non-Hodgkin lymphomas originating from the brain, meninges or spinal cord [1]. PDL are defined as an extra nodal lymphoma in the absence of systemic disease. Primary dural lymphomas (PDL) are very rare and are in general low-grade B cell lymphomas in anatomopathological examination [2]. The incidence of PCNSL is higher in immunocompromised patients, whereas PDL is seen in immune competent patients. PDL is rare and very few cases are reported in the literature that doesn't exceed the fifteen cases [1].

Our case exposes a PDL in a immune competent patient with small cell B lymphoma objectified in immunohistochemical examination.

PDL arises from the dura mater and belongs to the low-grade B-cell marginal zone group of tumors which is non-specific for CNS pathologies which are mostly high-grade diffuse large B-cell lymphomas [1].

PCNSL is a chemo sensitive and radiosensitive tumors. PDL has a better prognosis than PCNSL. Chemotherapy is used as a first-line treatment in dural lymphomas 7,8: chemotherapy drugs reach the dural tumor without passing the blood-brain barrier [2].

PDL tends to respond positively to surgery and radiotherapy. They are preferable choices due to its indolent character and high radio sensitivity but requires relatively low doses of radiotherapy [4,5]. Although there are limited reports and PDL is quite rare [1].

PDL has a better prognosis than other PCNSLs [2]. Without treatment, the prognosis for PCNSL is dismal (median survival of 1.5 months after diagnosis). Standard of care therapy with high dose methotrexate (MTX) in combination with other chemotherapy agents (rituximab, cytarabine) followed by whole brain radiation greatly improves overall survival up to a median of three years [3].

## 4 Conclusion

Primary dural lymphoma are a very rare type of central nervous tumor. Its radiologic aspect can lead to misdiagnosis in the radiological examination. Anatomopathological examination is the key to the diagnosis. Surgery associated with chemo and radiotherapy can improve the prognosis, which remains poor.

## Financial disclosure

The authors declared that this study has received no financial support.

## Ethical approval

Written informed consent was obtained from the patient for publication of this case report and accompanying images. A copy of the written consent is available for review by the Editor-in-Chief of this journal on request.

## Provenance and peer review

Not commissioned, externally peer-reviewed.

## Sources of funding

None.

## Consent

Written informed consent for publication of their clinical details and/or clinical images was obtained from the patient.

## Ethical approval

Ethical approval has been exempted by our institution.

## Research Registration Unique Identifying Number (UIN)

None.

## The trial registry number – ISRCTN

None.

## Author contributions

Jihane Saidy: writing the paper.

Abderrazzak Bertal: writing the paper.

Saad Hmada: writing the paper.

Nidal Aamara: writing the paper.

Yassine Tahrir: writing the paper and Corresponding author.

Sarah Moukhlis: writing the paper.

Mohamed Karkouri: Correcting the paper.

Abdessamad Naja: Correcting the paper.

Abdelhakim Lakhdar: Correcting the paper.

## Guarantor

Saidy Jihane.

## Declaration of competing interest

The authors declare having no conflicts of interest for this article.

## References

[1] Primary Dural Lymphoma Mimicking Meningioma: a Clinical and Surgical Case Report Marina Raguž1,*, Yannick Mudrovˇci'c1, Domagoj Dlaka1, Fadi Almahariq1,Dominik Romi'c1, Čedna Tomasovi'c-Loňcari'c2, Danko Müller2,Petar Mařcinkovi 'c1, Anđelo Kaštelaňci'c1, and Darko Chudy1. 10.1093/jscr/rjy189 PMC6077807 30093991

[2] Subdural B Cell Lymphoma Imaging Features, Histopathology. Literature Review Naga Varaprasad Vemuri, Lakshmi Sudha Prasanna Karanam, L. Rambabu, V.S.N. Rao, Kalyan, G. Sateesh. 10.1177/197140091302600605 PMC4202871 24355181

[3] Primary Central Nervous System Lymphoma Presenting as Chronic Subdural Hematoma: Case Report and Review of the Literature Alexa Semonche , Pablo Gomez , John Paul G. Kolcun , Roberto J. Perez-Roman , Robert M. Starke. 10.7759/cureus.7043 PMC7083252 32211275

[4] Iwamoto F.M., Abrey L.E. (2006). Primary dural lymphomas: a review. Neurosurg. Focus.

[5] Iwamoto F.M., DeAngelis L.M., Abrey L.E. (2006). Primary dural lymphomas: a clinicopathologic study of treatment and outcome in eight patients. Neurology.

[6] Taylor J.W., Flanagan E.P., O'Neill B.P., Siegal T., Omuro A., Deangelis L. (2013). Primary leptomeningeal lymphoma. Neurology.

[7] Agha R.A., Franchi T., Sohrabi C., Mathew G. (2020). For the SCARE Group, the SCARE 2020 guideline: updating consensus surgical CAse REport (SCARE) guidelines. Int. J. Surg..

